# Microstructure and Flux Pinning of Reacted-and-Pressed, Polycrystalline Ba_0.6_K_0.4_Fe_2_As_2_ Powders

**DOI:** 10.3390/ma12132173

**Published:** 2019-07-06

**Authors:** Michael R. Koblischka, Anjela Koblischka-Veneva, Jörg Schmauch, Masato Murakami

**Affiliations:** 1 Experimental Physics, Saarland University, P.O. Box 151150, D-66044 Saarbrücken, Germany; 2 Superconducting Materials Laboratory, Department of Materials Science and Engineering, Shibaura Institute of Technology, Tokyo 135-8548, Japan

**Keywords:** iron-based superconductors, critical currents, flux pinning, microstructure

## Abstract

The flux pinning properties of reacted-and-pressed Ba_0.6_K_0.4_Fe_2_As_2_ powder were measured using magnetic hysteresis loops in the temperature range 20 K ≤ *T* ≤ 35 K. The scaling analysis of the flux pinning forces ($F_{p}=j_{c}\times B$, with $j_{c}$ denoting the critical current density) following the Dew-Hughes model reveals a dominant flux pinning provided by normal-conducting point defects (δl-pinning) with only small irreversibility fields, $H_{\mathrm{irr}}$, ranging between 0.5 T (35 K) and 16 T (20 K). Kramer plots demonstrate a linear behavior above an applied field of 0.6 T. The samples were further characterized by electron backscatter diffraction (EBSD) analysis to elucidate the origin of the flux pinning. We compare our data with results of Weiss et al. (bulks) and Yao et al. (tapes), revealing that the dominant flux pinning in the samples for applications is provided mainly by grain boundary pinning, created by the densification procedures and the mechanical deformation applied.

## 1. Introduction

The discovery of iron-based superconductors (IBS) did not only initiate new research on the origin of superconductivity but also boosted new research on materials for applications [1]. Here, mainly three IBS compounds are important, the 1111-type (*R*OFeAs with *R* = denoting a rare earth element) materials, the 122-compounds (*A*Fe_2_As_2_ with *A* = alkaline earth) and the very simple iron-chalcogenides of the 11-type (e.g., FeSe). From all these compounds, thin films, as well as tapes and bulk samples, were already produced [1,2,3].

The compound Ba_0.6_K_0.4_Fe_2_As_2_ of the 122-family (hereafter abbreviated as 122) offers certain advantages as compared to cuprates, MgB_2_ and other IBS materials. The superconducting transition temperature, $T_{c}$, is higher than that of FeSe and it is less anisotropic than the 1111 compounds. Regarding possible applications, the 122 compounds can operate at higher magnetic fields than MgB_2_ [2], are less prone to flux jumps, are robust to impurity doping [3], and the misalignment of the grains can be higher than in cuprates when producing wires or bulks. These advantages were already manifested in several efforts to produce 122-based wires/tapes (see, e.g., the recent review of Yao et al. [4]), and trapped fields above 1 T were already recorded in bulk, HIP (hot isostatic pressed)-processed 122 samples [5]. On the other hand, the 122 material demonstrates several unique properties, e.g., it undergoes an isostructural transition under pressure [6,7,8,9], from which important information on the nature of superconductivity can be obtained.

In a previous review article, the flux pinning properties ($F_{p}=j_{c}\times B$, with $j_{c}$ denoting the critical current density, and $B=\mu_{0}H_{a}$), and especially, the scaling properties of the flux pinning forces of various IBS materials were discussed [10]. The 122 compounds analyzed showed a tendency towards dominant flux pinning of the δl-type, and a contribution of $\delta T_{c}$-pinning could not be excluded (that is, the peak position of the scaling diagrams ranged between 0.25 and 0.5). For the tapes, a further improvement of densification and deformation is an essential issue creating continuous current flow via many grain boundaries (GBs), and also, flux pinning may be provided by GBs as in the case of MgB_2_. This was discussed in detail in several publications [11,12,13,14]. The recent advances in $j_{c}$ of tapes [4] and bulk materials [5,15,16] are, therefore, due to these improved sample preparation routes.

Based on these developments, in the present contribution, we discuss the flux pinning properties measured on reacted-and-pressed 122 powder samples, and analyze the microstructural properties of the 122 grains by means of electron backscatter diffraction (EBSD). This combination of detailed microstructural analysis and magnetic data has already been proven to be very effective in previous publications [17,18]. Furthermore, we compare our data with recent data of 122 bulks and tapes.

## 2. Experimental Procedures

The Ba_0.6_K_0.4_Fe_2_As_2_ (122) powders were prepared using stoichiometric mixtures of the elements (purities > 99.9%) in alumina crucibles, which were sealed in silica tubes in an argon atmosphere. To prevent the evaporation of potassium during heat treatment, alumina inlays were employed. The mixtures were heated up to 600 °C (heating rate 50 °C/h). This temperature was maintained for 15 h. The reaction products were ground again and homogenized, sealed in a silica tube, and subjected to heat treatment at 650 °C for 15 h. Then, the mixtures were ground and homogenized and cold-pressed into pellets. Finally, the samples were sintered at 750 °C (20 h). The samples were furnace-cooled down to room temperature. The potassium evaporation caused only small fractions of the impurity phase FeAs (<3.5%), which was detected by X-ray analysis (Cu-K_*α*1_-radiation). The lattice parameters, the Ba/K atomic ratio, and the crystal purity of the 122 powders were already reported in [19,20,21]. Finally, to obtain the pellets studied here, the powders were again cold-pressed into pellets with 5 mm diameter. The X-ray data and the description of the preparation of ex-situ 122 tapes manufactured from the same powders are given in [21].

For the microstructural analysis, the sample surfaces were mechanically polished in dry conditions using various 3M imperial lapping sheets down to 0.25 µm roughness. A final polishing step using a colloidal silica solution (Struers water-free OP-AA solution) with a grain size of 25 nm was carried out. Details of the polishing procedure were already described in detail [22,23]. For the EBSD analysis, the surfaces of the samples were further subjected to low-angle argon ion-polishing (5 keV, 5 min) in order to improve the image quality of the Kikuchi patterns further. This procedure was described already for the investigation of multiferroic samples in [24,25].

The EBSD analysis was performed using a JEOL 7000F SEM microscope equipped with an orientation imaging analysis unit (EDAX Inc., OIM Analysis™) [26]. The Kikuchi patterns were generated at an acceleration voltage of 15 kV, and were recorded by means of a DigiView camera system. Automated EBSD mappings were carried out using step sizes down to 50 nm. The working distance was set to 15 mm.

Magnetic measurements were performed using a Quantum Design MPMS3 SQUID system with ±7 T applied field, and Quantum Design physical property measurement system (PPMS) systems equipped either with an extraction magnetometer or with a vibrating sample magnetometer option (±7 T and ±9 T applied magnetic field). In all cases, the field was applied perpendicular to the sample surface. The field sweep rate was always 0.36 T/min. From the magnetization data, the critical current densities, $j_{c}$, were evaluated using the extended Bean approach for rectangular samples [27] and the extensions described in [28,29,30]. The irreversibility lines were determined using a criterion of 100 A/cm^2^ from the obtained measurements, except the literature data [16], where extrapolation from the $j_{c}\left(H\right)$ graphs was employed [31].

## 3. Results and Discussion

**Figure 1:**
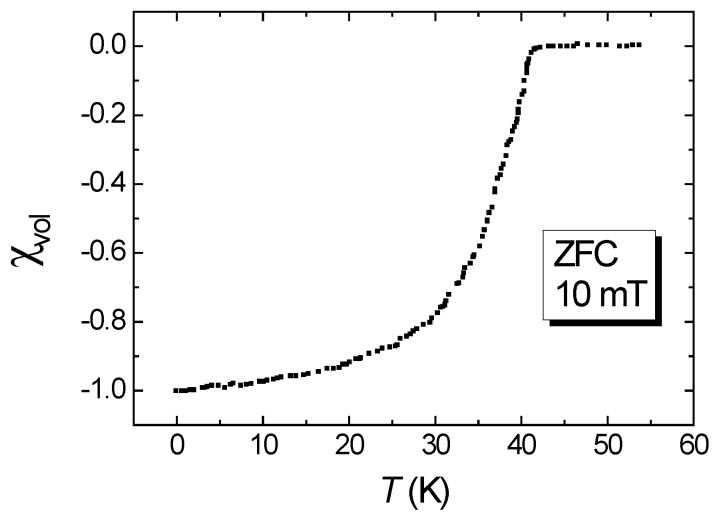
DC volume susceptibility, $\chi_{\mathrm{vol}}$, as function of temperature measured in a zero-field cooled (ZFC) condition (applied field 10 mT).

Figure 1 presents a DC susceptibility measurement of the reacted-and-pressed 122 powder sample in a zero-field cooled (ZFC) condition with an applied field of 10 mT. The transition temperature, $T_{c}$, was found to be 38.5 K and a relatively sharp superconducting transition was observed.

**Figure 2:**
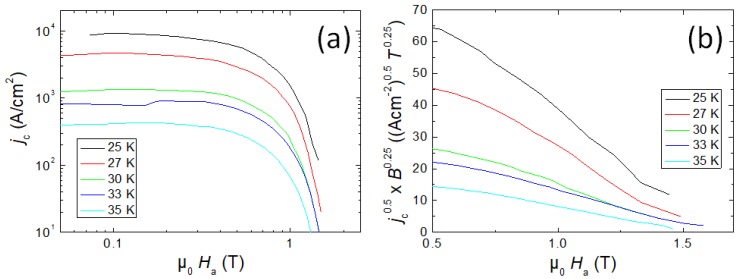
(**a**) double logarithmic plot of $j_{c}$ as a function of the applied field, $\mu_{0}H_{a}$; (**b**) Kramer plot of the same data set.

Figure 2 gives a double logarithmic plot of the critical current density, $j_{c}$, as a function of the applied field, $H_{a}$, in the temperature range 25 K to 35 K. Note that this current density is a true intra-grain current density only, as in the reacted-and-pressed powder sample no contribution of a transport current across the grain boundaries is expected. The flat part at low fields corresponds to single vortex pinning, which is then followed by a narrow regime with an intermediate curvature, and then a steep decrease towards the irreversibility is seen which is dominated by flux-flow. An extrapolation of this plot towards the 100 A/cm^2^-line gives information on the irreversibility fields used for the pinning force scaling below. The nominal values of $j_{c}$ obtained for the reacted-and-pressed powder sample of 10^4^ A/cm^2^ at 25 K and self-field are comparable to those of polycrystalline, bulk FeSe [32,33], but are clearly smaller than those of ex-situ powder-in-tube tapes produced using the same 122 powder. Here, a current density of 3 × 10^4^ A/cm^2^ was obtained at 4.2 K, using transport measurement on a 10 cm-long piece of tape with a filling factor of 30% [21].

To obtain more information about the flux pinning properties, Kramer plots, i.e., $j_{c}^{0.5}\times B^{0.25}$ versus the applied field, $B=\mu_{0}H_{a}$, are employed [34]. The advantage here is that the information obtained is free of uncertainties such as the determination of $H_{\mathrm{irr}}$. This is especially important when comparing data of different origins, i.e., unknown measurement parameters such as the field sweep rate. In Figure 2b, the Kramer plot for the same dataset is presented. At applied fields above 0.6 T, all curves are found to be quasi linear. An extrapolation of these curves yields information about the upper critical field, $H_{c2}$. It is obvious that $H_{c2}$ obtained in this way is always larger than the irreversibility fields, as shown below.

**Figure 3:**
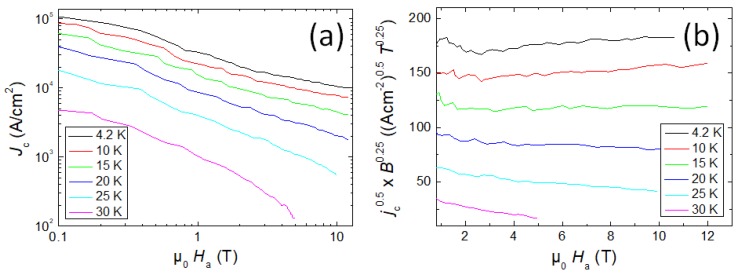
(**a**) double logarithmic plot of $j_{c}$ as a function of the applied field, $\mu_{0}H_{a}$; (**b**) Corresponding Kramer plot of the same data set.

For comparison of our data with those of a fully-reacted pnictide sample of the same composition, Figure 3a shows a double-log plot of the $j_{c}$-data by Weiss et al. [16] plotted in a similar manner to Figure 2. It is clearly visible that the critical current density obtained here is much higher, and the field dependence is very different, indicating high irreversibility fields. The magneto-optic images of flux penetration presented in [16] demonstrate that a homogeneous flux front penetrates the sample when applying external magnetic fields. This manifests as well-developed grain coupling, so the intergranular critical current density in this sample is high, and is clearly the predominant contribution to the overall $j_{c}$ measured with the magnetization data. Furthermore, the much higher $H_{\mathrm{irr}}$ data reveal that the flux pinning created by the mechanical deformation is predominant even up to high applied fields. Figure 3b gives the corresponding Kramer plot. Here, linear sections are also obtained. Note that the low-temperature data show an increase with increasing field, which indicates that the applied field range is still lower than the maximum pinning force ($F_{p}=j_{c}\times B$). The 122 sample of Weiss et al. was treated using hot-isostatic pressing (HIP), resulting in a dense sample microstructure with only a minor number of voids present [16]. The comparison of the two data sets indicates that the improved $j_{c}$ and $H_{\mathrm{irr}}$ values are due to densification and deformation during the HIP process.

**Figure 4:**
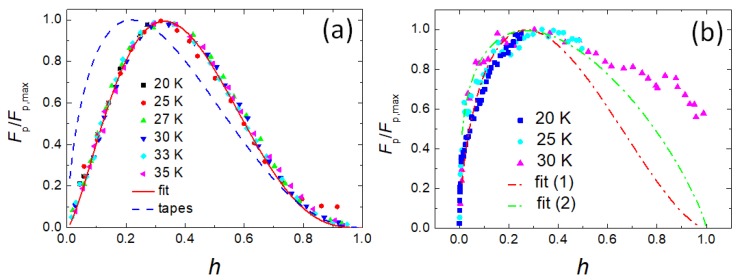
Pinning force scaling, $F_{p}/F_{p,\max}$ versus the reduced field, $h=H_{a}/H_{\mathrm{irr}}$, for the reacted-and-pressed 122 powder sample (**a**) and the HIP-processed 122 sample by Weiss et al. [16] (**b**). In (**a**), the best fit to the data is shown by a red line (−−). For comparison, the fit curve obtained on 122 tapes [3] is shown with a hatched blue line (− −). In (**b**), the two fits (1), (2) are attempts to fit the data by the DH model (for the parameters employed, see the main text).

In Figure 4a, the pinning force scaling following the approach of Dew-Hughes (DH) [35] of the reacted-and-pressed powder sample is shown. The obtained scaling is well developed, yielding a pinning function $Ah^{p}\times {\left(1-h\right)}^{q}$ with p = 1.5 and q = 3.05. The parameter *A* is not a real fit parameter, but defined via the condition $F_{p}/F_{p,\max}\left(h_{0}\right)$ = 1 [36]. Following the review on pinning force scaling of various IBS materials [10], this scaling is very similar to that of different single crystals and thin film samples of the 122 compounds. The resulting peak position, $h_{0}$, is obtained at 0.33, which indicates a dominant flux pinning provided by small normal-conducting inclusions.

Figure 4b illustrates the pinning force scaling of the data from Weiss et al. [16]. The irreversibility fields used for this scaling were obtained from extrapolations of the double-log plot as shown in Figure 3a, and finally, the data were adjusted by the scaling itself. For this procedure, we could only employ the data of 20, 25 and 30 K. For all other data, the measured field range is simply too small. Figure 4b demonstrates that it is possible to force the data to a scaling behavior, but it is difficult to obtain a proper fit to the data. The peak position, $h_{0}$, is only reached by the data taken at 25 K and 30 K. Here, it is clear that the scaling parameter *p* must be smaller than 1 to model the shape of the low-field part seen here, and the results of this attempt are depicted in Figure 4b using the red fit (1) with p = 0.6, q = 1.62 and the green fit (2) with p = 0.3, q = 0.81. Furthermore, the high-field tail of the 30 K data clearly deviates from any DH pinning function. For both fits, the peak is found at $h_{0}$ = 0.27, which is smaller than that of the reacted-and-pressed powder samples, but with a strong low-field side which does not reveal the typical curvature of the DH functions. The scaling parameters obtained here do not fit the DH model exactly, as the parameter *q* describes the type of pinning, and hence, takes only the values of 1 and 2 for superconducting and normal-conducting pinning sites, respectively. In Table 1, the values of *q* for the tapes are close to two, but deviations are seen for the reacted-and-pressed powder sample, as well as for fits (1) and (2) to the HIP-processed bulk sample. Values for *q* > 2 were often observed in 123-type cuprate superconductors [37,38] and also, in IBS materials [10], but a *q* < 0.5 (fit (2)) is outstanding, which could indicate a different pinning source; the DH function with p = 0.5 and q = 1 would describe magnetic volume pinning [35]. The parameter *p* can range between 0 and 2 and gives information about the extension of the flux pinning sites. A value of p = 0.5 corresponds to surface pinning (=GBs, 2D-pinning by extended defects), and p = 0 describes volume pinning, which would indicate that fit (2) describes pinning by GB clusters.

Furthermore, Figure 4a gives the result of the pinning force scaling of 122-tapes as determined by Huang et al. [39]. These authors obtained a good scaling in the range 25 K < T < 35 K with p = 0.64 and q = 2.3, yielding a peak position $h_{0}$ = 0.22. This clearly indicates that the predominant flux pinning is provided by the grain boundaries. A similar result was obtained by and Shabbir et al. [40], where p = 0.65 and q = 1.95 (0 GPa) and p = 0.8/q = 2 (0.7 GPa hydrostatic pressure) was obtained. These authors studied the application of hydrostatic pressure to densify the superconducting material. The change in the fitting parameters of the pinning force scaling (however, only measured at T = 24 K) reveals that the pressure induced more point defects into the material, which improve the flux pinning properties even further, thereby, a record high $j_{c}$ was obtained at 4.2 K.

**Figure 5:**
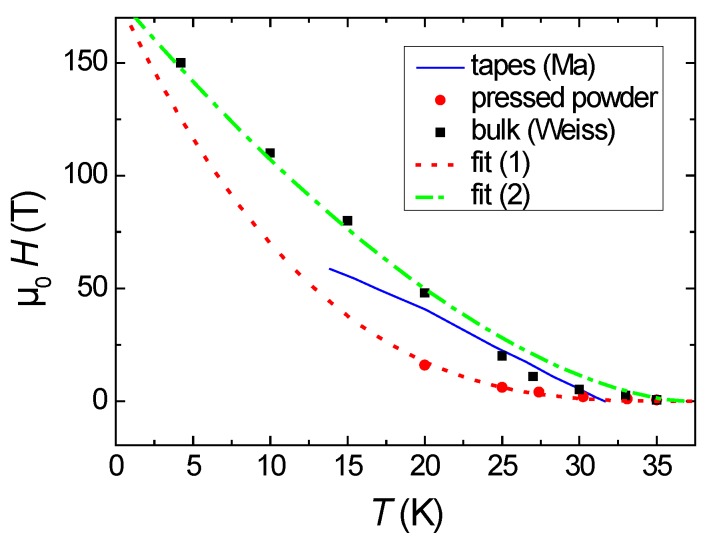
Irreversibility fields, $H_{\mathrm{irr}}$, for the reacted-and-pressed powder sample (●), tapes indicated by a blue line (−−) [39], and the HIP-treated bulk sample (■) of Weiss et al. [16]. Details of the fits (1) and (2) are described in the main text.

Figure 5 presents the irreversibility fields, $H_{\mathrm{irr}}$, obtained for the reacted-and-pressed powder sample (red bullets), the data extracted from the measurements of Weiss et al. (black squares) and the data from Huang et al. [39] (blue line) for 122 tapes. The comparison here is straightforward as all samples exhibit a similar value of $T_{c}$, in contrast to the comparison presented in [33]. The reacted-and-pressed 122 sample shows only small irreversibility fields in comparison with the other sample types (bulks, tapes). At temperatures close to $T_{c}$, the tape-data and those of Weiss are similar to each other, and at lower temperatures, the data of Weiss are much higher, which may also be due to the different measurement techniques (magnetic vs. transport). When fitting the data using the relation $H_{\mathrm{irr}}\left(T\right)=H_{0}{\left(1-T/T_{c}\right)}^{n}$ with n = 3, common for many 123-high-$T_{c}$ superconducting materials [41], we obtain a fit parameter $H_{0}$ = 180 T (fit (1)). To fit the data of Weiss et al., we can use also $H_{0}$ = 180 T, but *n* is changed to 1.65 (fit (2)). This behavior points again to the fact that the origin of flux pinning of the reacted-and-pressed 122 powder is similar to that in cuprate superconductors, especially YBa_2_Cu_3_O_7_ (YBCO), where a peak position of $h_{0}$ = 0.33 is also obtained. Thus, a weak collective pinning by point defects is predominant. However, in contrast to the YBCO compounds, the $j_{c}$ and $H_{\mathrm{irr}}$ values in the reacted-and-pressed powder sample are small, whereas both values are high in YBCO, even for polycrystalline samples. In the case of the 122 samples for applications that have underwent densification/mechanical treatments, a different origin of the flux pinning is revealed as indicated by the shift of the peak position, $h_{0}$, of the pinning force scaling diagram towards smaller values close to 0.2. This observation points to the strong role of the mechanical deformation [42] and densification processes to develop a strong flux pinning in the 122 materials.

The critical current densities measured in the reacted-and-pressed powder sample are truly intragrain current densities, as there is no current flow across the GBs. From the data presented here, we can conclude that the intragrain flux pinning is provided by a weak δl-pinning (small normal-conducting inclusions or defects), which also determines the small values of $H_{\mathrm{irr}}$ and $H_{c2}$. In contrast, the strong pinning and the large irreversibility fields found in the deformed (densified) 122 samples is provided by GB pinning, which neither exists in the reacted-and-pressed 122 powder samples nor in the 122 single crystals investigated in the literature. Furthermore, this GB pinning is also responsible for the high irreversibility fields of the 122 tapes and bulks. These observations are in contrast to the cuprate superconductors, and here, especially YBCO, where the intragranular current density of the grains is always high, and also dominates $H_{\mathrm{irr}}$.

Now, we turn to the microstructural analysis of the reacted-and-pressed powder sample by means of EBSD. This provides the possibility to compare the data with those obtained on 122 tapes already published in the literature [39,40].

**Figure 6:**
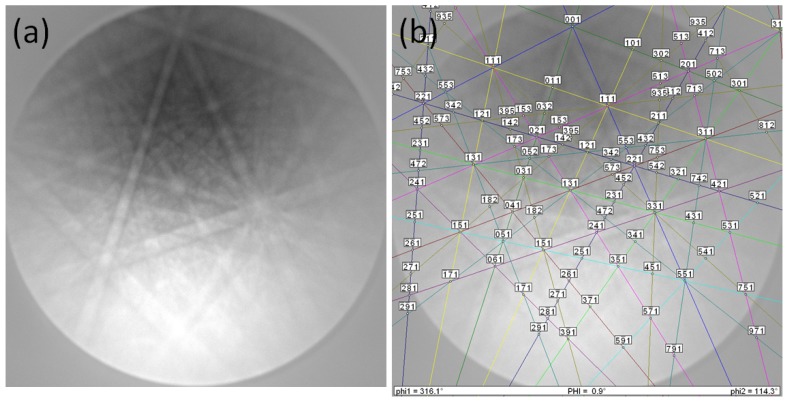
(**a**) Kikuchi pattern of the 122 compound and (**b**) its indexation (Image quality ∼6000, CI = 1). The resulting Euler angles are $\phi_{1}$ = 316.1°, Φ = 0.9° and $\phi_{2}$ = 114.3°.

Figure 6 presents the Kikuchi patterns of the 122 compound and the EBSD indexation. The resulting Euler angles are $\phi_{1}$ = 316.1°, Φ = 0.9° and $\phi_{2}$ = 114.3°. The image quality (IQ) is high at ∼6000, and the confidence index for the indexation is 1 (i.e., exact indexation).

**Figure 7:**
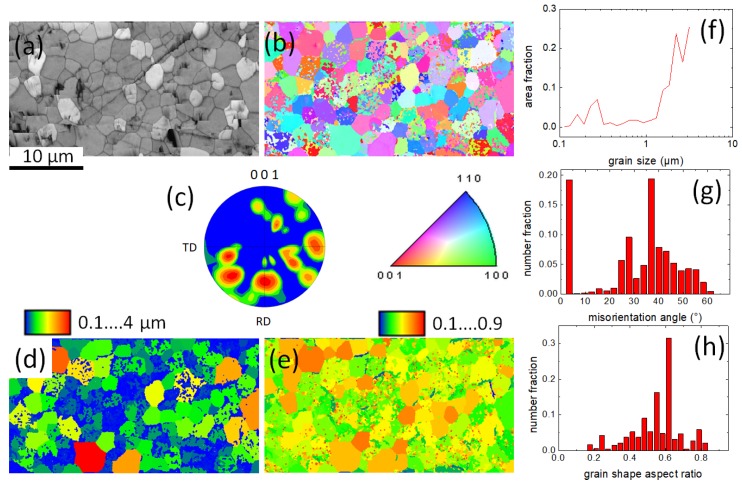
Electron backscatter diffraction (EBSD) analysis of the 122 reacted-and-pressed powder sample. (**a**) is an image quality (IQ) map; (**b**) gives the orientation mapping in the direction normal to the sample surface. The color code for this map is given in the stereographic triangle below the map; (**c**) presents the corresponding pole figure in the [001]-direction (i.e., normal to the sample surface, or ND); (**d**) is a grain size mapping, and (**e**) is a map of the grain size aspect ratio. The color codes for the maps (**d**,**e**) are indicated at the top of each map. The plots (**f**–**h**) illustrate details of the sample microstructure. (**f**) gives the statistics of the EBSD-determined grain size, (**g**) the distribution of the misorientation angles, and (**h**) the statistics of the grain shape aspect ratio. For details, see the main text.

In Figure 7a–g, EBSD mappings and dataplots are presented. Figure 7a is an image quality map, which resembles a backscattered electron image, but in EBSD conditions (70° tilt), of the sample. Here, we see that there are many large grains, but also a high number of small grains. There is a large amount of voids and cracks in the investigated sample area, typical for such a reacted-and-pressed powder sample. Figure 7b presents an orientation mapping in the direction normal to the sample surface (i.e., in [001]-direction or ND). The color code for this map is given in the stereographic triangle below. The result of the grain orientation mapping is also presented in the corresponding pole figure (c), showing several distinct maxima. The map (d) is a grain size map with grain sizes ranging from 0.1 µm (blue) to 4 µm (red). This is further illustrated in the corresponding grain size plot (f). The grain sizes range between 110 nm and 3.2 µm. The map (e) gives the distribution of the grain shape aspect ratio, $\gamma_{\mathrm{ar}}$. To obtain this information, the 122 grains in the orientation map (b) are encircled by ellipses by the EBSD software. The grain shape aspect ratio, $\gamma_{\mathrm{ar}}$, is defined as the length of the minor axis divided by the length of the major axis. The value of $\gamma_{\mathrm{ar}}$ ranges between 0 and 1, with higher values indicating similar lengths of the axes [26]. The color code for this map is indicated on top of it. The plot (g) gives the statistics of the EBSD-determined misorientation angles (number fraction vs. misorientation angle). Here, a large number of small-angle misorientations is obtained, and also a maximum at ∼37°. Finally, the results of the grain shape aspect ratio analysis are presented in plot (h). The majority of the 122 grains are only slightly elliptic; grains with an elongated major axis $\gamma_{\mathrm{ar}}$ between 0.1 and 0.4 are quite rare in the investigated area. Comparing our results of the EBSD-analysis of the reacted-and-pressed powder sample to those of 122-tapes published in the literature [39,40], we see that the 122 grains in the tapes show a more elliptic grain aspect, which is a consequence of the deformation treatment applied. This is a remarkable result of the EBSD investigation, and is important for the processing of future 122 samples for applications. The development of the specific microstructure in the tape samples and that of HIP-processed bulks will, therefore, require more detailed investigations in the future to further improve the performance of the 122 superconductor samples.

The results obtained on the reacted-and-pressed powder sample corroborate that the mechanical deformation and densification processes (e.g., HIP, wire drawing and rolling) play an essential role in order to achieve the desired flux pinning properties, and not only the improvement of texture, such as in cuprate superconductors, where the intragrain critical current density ($j_{c}$(intra)) always plays the predominant role. Both the critical current densities (flux pinning forces) and the irreversibility fields develop their strength in the 122 samples upon occurrence of mechanical deformation processes, whereas the reacted-and-pressed 122 powder sample is found to behave much more similarly to single crystals of the same compound [10]. The comparison of the EBSD results on the reacted-and-pressed powder samples with that obtained on 122 tapes reveals changes in the grain shape that were induced by the processing. Therefore, to achieve even higher critical current densities (flux pinning forces), more efforts to study the microstructural changes in the mechanical deformation processes of the 122-grains are required.

## 4. Conclusions

To conclude, we have presented magnetic and microstructural data of a reacted-and-pressed 122 powder sample and performed a comparison of our results with the literature data of a HIP-processed 122 bulk sample and 122 tapes. The obtained critical current densities (which are intragrain only) and the irreversibility fields of the reacted-and-pressed powder sample are much lower as compared to the bulks and tapes. The pinning force scaling of the reacted-and-pressed powder sample is well developed, with a dominant pinning provided by normal-conducting small inclusions (peak position at 0.33), similar to 122 single crystals. This is in contrast to the results obtained on HIP-processed 122 bulks and 122 tapes, where much lower peak positions in the pinning force scaling diagrams are obtained (0.27 and 0.22). This points to a dominant flux pinning provided by grain boundaries, so that $j_{c}\left(\mathrm{inter}\right)>j_{c}\left(\mathrm{intra}\right)$. Therefore, the mechanical deformation and densification processes induced in manufacturing samples for applications are essential to achieve high critical currents and high irreversibility fields in the 122 samples.

## Figures and Tables

**Table 1:** Fitting parameters and peak positions of the DH scaling of various 122 sample types. For the references, see the main text.

Sample	*p*	*q*	*h*_0_	Remarks
reacted-and-pressed	1.5	3.05	0.33	good scaling, similar to 122 crystals
HIP-processed bulk				
fit (1)	0.6	1.62	0.27	fit close to the low-field data at all *T*
fit (2)	0.3	0.81	0.27	steeper fit, 20 K data deviate clearly
tapes				
Huang	0.64	2.3	0.22	good scaling, 20 K ≤ *T* ≤ 32 K
Shabbir (0 GPa)	0.65	1.95	0.25	*T* = 24 K
Shabbir (0.7 GPa)	0.8	2	0.29	*T* = 24 K, hydrostatic pressure applied

## References

[1] Hosono H., Yamamoto A., Hiramatsu H., Ma Y. (2018). Recent advances in iron-based superconductors toward applications. Mater. Today.

[2] Gurevich A. (2011). Iron-based superconductors at high magnetic fields. Rep. Prog. Phys..

[3] Ma Y. (2012). Progress in wire fabrication of iron-based superconductors. Supercond. Sci. Technol..

[4] Yao C., Ma Y. (2019). Recent breakthrough development in iron-based superconducting wires for practical applications. Supercond. Sci. Technol..

[5] Weiss J.D., Yamamoto A., Polyanskii A.A., Richardson R.B., Larbalestier D.C., Hellstrom E.E. (2015). Demonstration of an iron-pnictide bulk superconducting magnet capable of trapping over 1 T. Supercond. Sci. Technol..

[6] Uhoya W., Stemshorn A., Tsoi G., Vohra Y.K., Sefat A.S., Sales B.C., Hope K.M., Weir S.T. (2010). Collapsed tetragonal phase and superconductivity of BaFe_2_As_2_ under high pressure. Phys. Rev. B.

[7] Mittal R., Mishra S.K., Chaplot S.L., Ovsyannikov S.V., Greenberg E., Trots D.M., Dubrovinsky L., Su Y., Brueckel T., Matsuishi S. (2011). Ambient- and low-temperature synchrotron x-ray diffraction study of BaFe_2_As_2_ and CaFe_2_As_2_ at high pressures up to 56 GPa. Phys. Rev. B.

[8] Nakajima Y., Wang R., Metz T., Wang X., Wang L., Cynn H., Weir S.T., Jeffries J.R., Paglione J. (2015). High-temperature superconductivity stabilized by electron-hole interband coupling in collapsed tetragonal phase of KFe_2_As_2_ under high pressure. Phys. Rev. B.

[9] Ptok A., Sternik M., Kapcia K.J., Piekarz P. (2019). Structural, electronic, and dynamical properties of the tetragonal and collapsed tetragonal phases of KFe_2_As_2_. Phys. Rev. B.

[10] Koblischka M.R., Muralidhar M. (2016). Pinning force scaling analysis of Fe-based high-*T_c_* superconductors. Int. J. Mod. Phys. B.

[11] Katase T., Ishimaru Y., Tsukamoto A., Hiramatsu H., Kamiya T., Tanabe K., Hosono H. (2011). Advantageous grain boundaries in iron pnictide superconductors. Nat. Commun..

[12] Kametani F., Li P., Abraimov D., Polyanskii A.A., Yamamoto A., Jiang J., Hellstrom E.E., Gurevich A., Larbalestier D.C., Ren Z.A. (2009). Intergrain current flow in a randomly oriented polycrystalline SmFeAsO_0.85_ oxypnictide. Appl. Phys. Lett..

[13] Hecher J., Baumgartner T., Weiss J.D., Tarantini C., Yamamoto A., Jiang J., Hellstrom E.E., Larbalestier D.C., Eisterer M. (2016). Small grains: A key to high-field applications of granular Ba-122 superconductors?. Supercond. Sci. Technol..

[14] Iida K., Hänisch J., Tarantini C. (2018). Fe-based superconducting thin films on metallic substrates: Growth, characteristics, and relevant properties. Appl. Phys. Rev..

[15] Weiss J.D., Tarantini C., Jiang J., Kametani F., Polyanskii A.A., Larbalestier D.C., Hellstrom E.E. (2012). High intergrain critical current density in fine-grain (Ba_0.6_K_0.4_)Fe_2_As_2_ wires and bulks. Nat. Mater..

[16] Weiss J.D., Jiang J., Polyanskii A.A., Hellstrom E.E. (2013). Mechanochemical synthesis of pnictide compounds and superconducting Ba_0.6_K_0.4_Fe_2_As_2_ bulks with high critical current density. Supercond. Sci. Technol..

[17] Koblischka-Veneva A., Koblischka M.R., Qu T., Han Z., Mücklich F. (2008). Texture analysis of monofilamentary, Ag-sheathed (Pb,Bi)_2_Sr_2_Ca_2_Cu_3_O_*x*_ tapes by electron backscatter diffraction (EBSD). Physica C.

[18] Koblischka-Veneva A., Koblischka M.R., Schmauch J., Inoue K., Muralidhar M., Berger K., Noudem J. (2016). EBSD analysis of MgB_2_ bulk superconductors. Supercond. Sci. Technol..

[19] Wiesenmayer E., Luetkens H., Pascua G., Khasanov R., Amato A., Potts H., Banusch B., Klauss H.-H., Johrendt D. (2011). Microscopic Coexistence of Superconductivity and Magnetism in Ba_1−*x*_K_*x*_Fe_2_As_2_. Phys. Rev. Lett..

[20] Wiesenmayer J.E. Dissertation LMU München, Germany. https://edoc.ub.uni-muenchen.de/18509/1/Wiesenmayer_Josef_Erwin.pdf.

[21] Malagoli A., Wiesenmayer E., Marchner S., Johrendt D., Genovese A., Putti M. (2015). Role of heat and mechanical treatments in the fabrication of superconducting Ba_0.6_K_0.4_Fe_2_As_2_ ex situ powder-in-tube tapes. Supercond. Sci. Technol..

[22] Koblischka M.R., Koblischka-Veneva A. (2013). Applications of the electron backscatter diffraction technique to ceramic materials. Phase Transit..

[23] Koblischka M.R., Koblischka-Veneva A., Alguero M., Gregg J.M., Mitoseriu L. (2016). Advanced Characterization of Multiferroic Materials by Scanning Probe Methods and Scanning Electron Microscopy. Nanoscale Ferroelectrics and Multiferroics.

[24] Koblischka-Veneva A., Koblischka M.R., Schmauch J., Chen Y., Harris V.G. (2010). EBSD analysis of the microtexture of Ba-hexaferrite samples. J. Phys. Conf. Ser..

[25] Koblischka-Veneva A., Koblischka M.R., Chen Y., Harris V.G. (2009). Analysis of Grain Shape and Orientation in BaFe_12_O_19_-Ferrites Using Electron Backscatter Diffraction (EBSD). IEEE Trans. Magn..

[26] (2018). Orientation Imaging Microscopy (OIM Analysis™).

[27] Wiesinger H.P., Sauerzopf F.M., Weber H.W. (1992). On the calculation of *J_c_* from magnetization measurements on superconductors. Physica C.

[28] Gokhfeld D.M., Balaev D.A., Petrov M.I., Popkov S.I., Shaykhutdinov K.A., Val’kov V.V. (2011). Magnetization asymmetry of type-II superconductors in high magnetic fields. J. Appl. Phys..

[29] Gokhfeld D.M. (2013). Secondary Peak on Asymmetric Magnetization Loop of Type-II Superconductors. J. Supercond. Novel Magn..

[30] Gokhfeld D.M. (2019). The circulation radius and critical current density in type-II superconductors. Tech. Phys. Lett..

[31] Sun Y., Pyon S., Tamegai T., Kobayashi R., Watashige T., Kasahara S., Matsuda Y., Shibauchi T., Kitamura H. (2015). Enhancement of critical current density and mechanism of vortex pinning in H^+^-irradiated FeSe single crystal. Appl. Phys. Exp..

[32] Karwoth T., Furutani K., Koblischka M.R., Zeng X.L., Wiederhold A., Muralidhar M., Murakami M., Hartmann U. (2018). Electrotransport and magnetic measurements on bulk FeSe superconductors. J. Phys. Conf. Ser..

[33] Koblischka-Veneva A., Koblischka M.R., Berger K., Nouailhetas Q., Douine B., Muralidhar M., Murakami M. (2019). Comparison of Temperature and Field Dependencies of the Critical Current Densities of Bulk YBCO, MgB_2_, and Iron-Based Superconductors. IEEE Trans. Appl. Supercond..

[34] Kramer E.J. (1973). Scaling laws for flux pinning in hard superconductors. J. Appl. Phys..

[35] Dew-Hughes D. (1974). Flux pinning mechanisms in type-II superconductors. Philos. Mag..

[36] Koblischka M.R. (1997). Pinning in bulk high-*T_c_* superconductors. Inst. Phys. Conf. Ser..

[37] Koblischka M.R., van Dalen A.J., Higuchi T., Yoo S.I., Murakami M. (1998). Analysis of pinning in NdBa_2_Cu_3_O_7−*δ*_ superconductors. Phys. Rev. B.

[38] Koblischka M.R., Muralidhar M. (2014). Pinning force scaling and its analysis in the LRE-123 ternary compounds. Physica C.

[39] Huang H., Yao C., Dong C.H., Zhang X.P., Wang D.L., Cheng Z., Li J.Q., Awaji S., Wen H.H., Ma Y.W. (2018). High transport current superconductivity in powder-in-tube Ba_0.6_K_0.4_Fe_2_As_2_ tapes at 27 T. Supercond. Sci. Technol..

[40] Shabbir B., Huang H., Yao C., Ma Y.W., Dou S.X., Johansen T.H., Hosono H., Wang X.L. (2017). Evidence for superior current carrying capability of iron pnictide tapes under hydrostatic pressure. Phys. Rev. Mater..

[41] Altin E., Gokhfeld D.M., Demirel S., Oz E., Kurt F., Altin S., Yakinci M.E. (2014). Vortex pinning and magnetic peak effect in Eu(Eu,Ba)_2.125_Cu_3_O_*x*_. J. Mater. Sci. Mater. Electron..

[42] Han Z., Skov-Hansen P., Freltoft T. (1997). The mechanical deformation of superconducting BiSrCaCuO/Ag composites. Supercond. Sci. Technol..

